# Polyphosphate Kinase Is Required for the Processes of Virulence and Persistence in Acinetobacter baumannii

**DOI:** 10.1128/spectrum.01230-22

**Published:** 2022-07-05

**Authors:** Hongfa Lv, Yonglin Zhou, Baichen Liu, Jian Guan, Peng Zhang, Xuming Deng, Dan Li, Jianfeng Wang

**Affiliations:** a Department of Respiratory Medicine, Center for Pathogen Biology and Infectious Diseases, Key Laboratory of Organ Regeneration and Transplantation of the Ministry of Education, The First Hospital of Jilin University, Changchun, China; b State Key Laboratory for Zoonotic Diseases, College of Veterinary Medicine,Jilin University, Changchun, China; c The Second Bethune Clinical Medical College of Jilin University, Changchun, China; d Department of Thoracic Surgery, The First Hospital of Jilin University, Changchun, China; Emory University School of Medicine

**Keywords:** *A. baumannii*, polyphosphate kinase 1, polyphosphate, virulence, persistence

## Abstract

Acinetobacter baumannii, one of the most successful bacteria causing severe nosocomial infection, was identified as a top-priority pathogen by the WHO. Thus, genetic manipulations to clarify the potential targets for fighting A. baumannii resistance and virulence are vital. Polyphosphate (polyP) kinase (PPK) is conserved in nearly all bacteria and is responsible for polyP formation, which is associated with bacterial pathogenicity and antibiotic resistance. In this study, *ppk1*-deficient (Δ*ppk1*::Apr), *ppk1*-complemented (Δ*ppk1*::Apr/PJL02-*ppk1*), and wild-type strains of A. baumannii ATCC 17978 were used to determine the influence of PPK1 on A. baumannii virulence and persistence mainly by polyP quantification, surface motility, biofilm formation, and bacterial persistence assays. Our work found that PPK1 is indispensable for polyP formation *in vivo* and that the motility of the PPK1-deficient strain was significantly impaired due to the lack of a pilus-like structure typically present compared with the complemented and wild-type strains. The deficiency of PPK1 also inhibited the biofilm formation of A. baumannii and decreased bacterial persistence under stimuli of high-concentration ampicillin (Amp) treatment, H_2_O_2_ stress, heat shock, and starvation stress. Furthermore, *ppk1*-deficient bacterium-infected mice showed a significantly reduced bacterial load and a decreased inflammatory response. However, complementation with PPK1 effectively rescued the impaired virulence and persistence of *ppk1*-deficient A. baumannii. In addition, metabonomic analysis revealed that PPK1 was associated with glycerophospholipid metabolism and fatty acid biosynthesis. Taken together, our results suggest that targeting PPK1 to control A. baumannii pathogenicity and persistence is a feasible strategy to fight this pathogen.

**IMPORTANCE** A. baumannii was identified as a top-priority pathogen by the WHO due to its antibiotic resistance. Meanwhile, the pathogenicity of A. baumannii mediated by several vital virulence factors also cannot be ignored. Here, the role of PPK1 in A. baumannii was also explored. We found that the motility ability and biofilm formation of a PPK1-deficient strain were significantly impaired. Furthermore, PPK1 was essential for its persistence maintenance to resist stimuli of high-concentration Amp treatment, H_2_O_2_ stress, heat shock, and starvation stress. Metabonomic analysis revealed that PPK1 was associated with glycerophospholipid metabolism and fatty acid biosynthesis. In addition, *ppk1*-deficient bacterium-infected mice showed significantly reduced bacterial loads and a decreased inflammatory responses *in vivo*. Together, our results suggest that PPK1 is vital for A. baumannii pathogenicity and persistence.

## INTRODUCTION

Acinetobacter baumannii is an opportunistic nosocomial pathogen that causes ventilator-associated pneumonia as well as bloodstream infections in intensive care unit (ICU) patients. In recent years, A. baumannii has aroused widespread concern due to its virulence and persistence (1). The pathogenicity of A. baumannii is mediated mainly by several vital virulence factors that are associated with motility (2) and adhesion to and invasion of host cells (3) that collectively enable A. baumannii to successfully infect its host. Regarding drug resistance, A. baumannii is also considered an emerging health threat due to its multidrug-resistant characteristics (4). Infections caused by MDR (Multiple drug resistance) *A. baumannii* especially in carbapenem-resistant strains, is relevant to substantial mortality and medical care burdens (5). In addition, A. baumannii can combat various environmental stresses such as disinfection, osmotic challenge, acid stress, and oxidative stress. Therefore, simultaneous targeting of A. baumannii resistance and virulence represents a viable and effective strategy to treat A. baumannii infection.

Similar to ATP, inorganic polyphosphate (polyP) is essentially a phosphoanhydride consisting of tens to hundreds of phosphate residues (6). PolyP in bacterial cells is involved in bacterial virulence and resistance processes such as biofilm formation, energy metabolism, motility, stress defense, and bacterial survival in cells (7, 8). In eukaryotes, the role of polyP is related to blood clotting, apoptosis, DNA repair, inflammation, and amyloid fiber formation (9). Polyphosphate kinases (PPKs), including PPK1 and PPK2, are essential enzymes that participate in the polyP metabolism of bacteria and catalyze ATP to form polyP in a reversible way (10, 11). PPKs exist in a variety of microorganisms, including bacteria, yeast, algae, and even fungi, but they have not been discovered in mammalian cells (11), suggesting that future studies are needed to define the beneficial effect of PPKs on infections by different microorganisms and that PPKs represent ideal targets for treating bacterial infection without affecting the host.

Some phenotypic changes have also been observed in PPK-deficient strains. Inhibition of PPK1 enzymatic activity disrupted biofilm formation and reduced the pathogenicity of uropathogenic Escherichia coli (12). PPK2 mutants of Mycobacterium tuberculosis also displayed impaired biofilm formation and restored sensitivity to antibiotics (13). A. baumannii PPK1 was associated with biofilm formation and motility, which has been confirmed (14). However, that study was short of PPK1-deficient strains. In our study, we explored the function of PPK1 in A. baumannii ATCC 17978 in terms of virulence and persistence. A *ppk1*-deficient strain and its complemented strain were constructed for the first time in A. baumannii ATCC 17978. Furthermore, phenotypic changes, including biofilm formation, motility, metabolism, and persistence, among the wild type (WT), the *ppk1*-deficient strain, and the *ppk1*-complemented strain were evaluated. A mouse pneumonia model of A. baumannii ATCC 17978 infection was established to evaluate the role of PPK1 in A. baumannii infection. Overall, our results indicated that PPK1 represents a promising target for treating A. baumannii infection, as evidenced by the observation that PPK1 is essential for A. baumannii virulence and persistence.

## RESULTS

### Construction of *ppk1*-deficient and -complemented strains of A. baumannii ATCC 17978.

As shown in Fig. S1A in the supplemental material, the apramycin resistance gene was linked with the upstream and downstream homologous recombination DNA fragments of *ppk1*. Clones of the 1st, 3rd, 8th, 13th, 19th, and 20th percentiles were the correct singly exchanged clones (Fig. S1B). The *ppk1-*deficient mutant of A. baumannii strain ATCC 17978 strain was named Δ*ppk1*::Apr, in which the *ppk1* gene was successfully replaced by the apramycin resistance gene and could not be amplified from the correct clone (Fig. S1C and D). For the generation of complemented strains, the plasmid PJL02 linked with the *ppk1* gene was transformed into E. coli WM3064 and conjoined with Δ*ppk1*::Apr to construct the complemented strain, named Δ*ppk1*::Apr/PJL02-*ppk1*. As shown in Fig. S2, *ppk1* could be expressed with inductions with isopropyl-β-d-thiogalactopyranoside (IPTG) at less than 0.25 μg/mL. Thus, we successfully constructed Δ*ppk1*::Apr, a *ppk1-*deficient mutant of A. baumannii strain ATCC 17978, and Δ*ppk1*::Apr/PJL02-*ppk1*, a *ppk1-*complemented A. baumannii ATCC 17978 mutant.

### PPK1 is essential for polyP accumulation in bacterial cells of A. baumannii.

**FIG 1:**
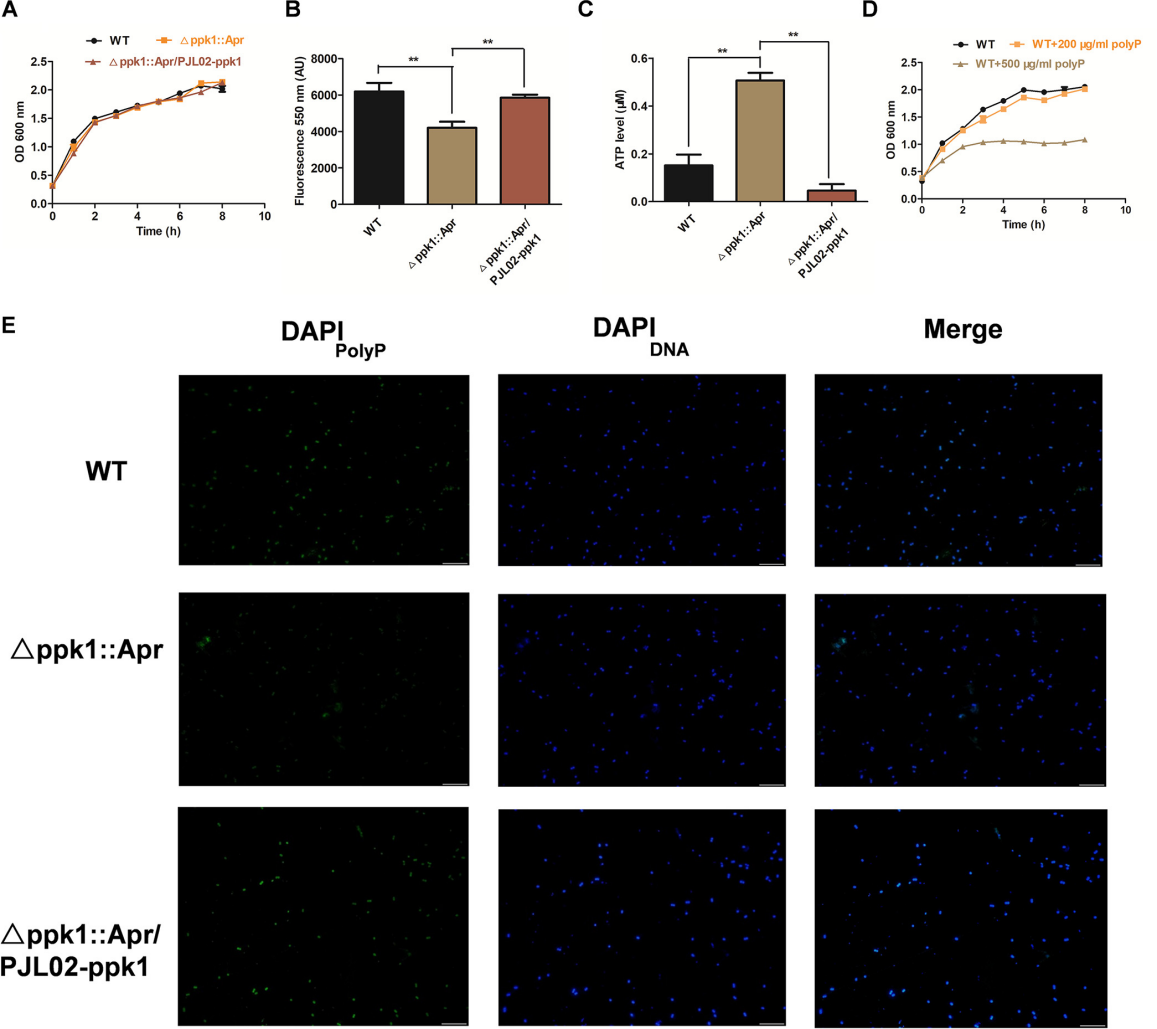
PPK1 is essential for polyP accumulation *in vivo*. (A) Growth curves of the WT and its derivative strains in LB broth. (B and C) PolyP level determinations (B) and intracellular ATP level measurements (C) of the WT, Δ*ppk1*::Apr, and Δ*ppk1*::Apr/PJL02-*ppk1* strains at logarithmic phase under MOPS low-phosphate minimal medium culture for 2 h. AU, arbitrary units. (D) Growth curves of the WT with different concentrations (0 μg/mL, 200 μg/mL, and 500 μg/mL) of polyP. (E) PolyP visualization of the WT, Δ*ppk1*::Apr, and Δ*ppk1*::Apr/PJL02-*ppk1* strains under a confocal microscope after MOPS low-phosphate minimal medium culture for 2 h.

The growth curves were analyzed to determine whether the deficiency of *ppk1* impairs the growth of A. baumannii. The results showed that the growth state of the *ppk1*-deficient strain (Δ*ppk1*::Apr) was similar to that of the WT or the complemented Δ*ppk1*::Apr/PJL02-*ppk1* strain, which indicated that the deletion of *ppk1* had no influence on the growth of A. baumannii (Fig. 1A). Furthermore, we explored the effect of the addition of polyP on bacterial growth. As shown in Fig. 1D, 200 μg/mL polyP had no effect on bacterial growth, and this polyP concentration was used for the following experiment.

PPK1, as an important polyP synthetase, has been shown to affect metabolism in Pseudomonas aeruginosa (13). Therefore, the polyP accumulation level was determined in our study. As expected, we found that the polyP level in the Δ*ppk1*::Apr strain was significantly reduced by 32.1% and 28.1% compared with those in the WT and Δ*ppk1*::Apr/PJL02-*ppk1* strains, respectively (Fig. 1B). In addition, previous studies indicated that decreased polyP levels could lead to high ATP accumulation under nutrient deprivation (15). Our results suggested that the intracellular ATP level of the Δ*ppk1*::Apr strain was also higher than that of the WT or Δ*ppk1*::Apr/PJL02-*ppk1* strain (Fig. 1C). Moreover, the polyP contents in the WT, Δ*ppk1*::Apr, and Δ*ppk1*::Apr/PJL02-*ppk1* strains were visualized by confocal microscopy (16). The fluorescence intensity of DNA (blue) was similar to that of the WT or Δ*ppk1*::Apr/PJL02-*ppk1* strain. as shown in Fig. 1E. However, the fluorescence intensity of polyP (green) in the Δ*ppk1*::Apr strain was lower than that in the WT or Δ*ppk1*::Apr/PJL02-*ppk1* strain. Taken together, our results indicated that PPK1 is essential for polyP accumulation in bacterial cells of A. baumannii.

### PPK1 is involved in the surface-associated motility of A. baumannii.

**FIG 2:**
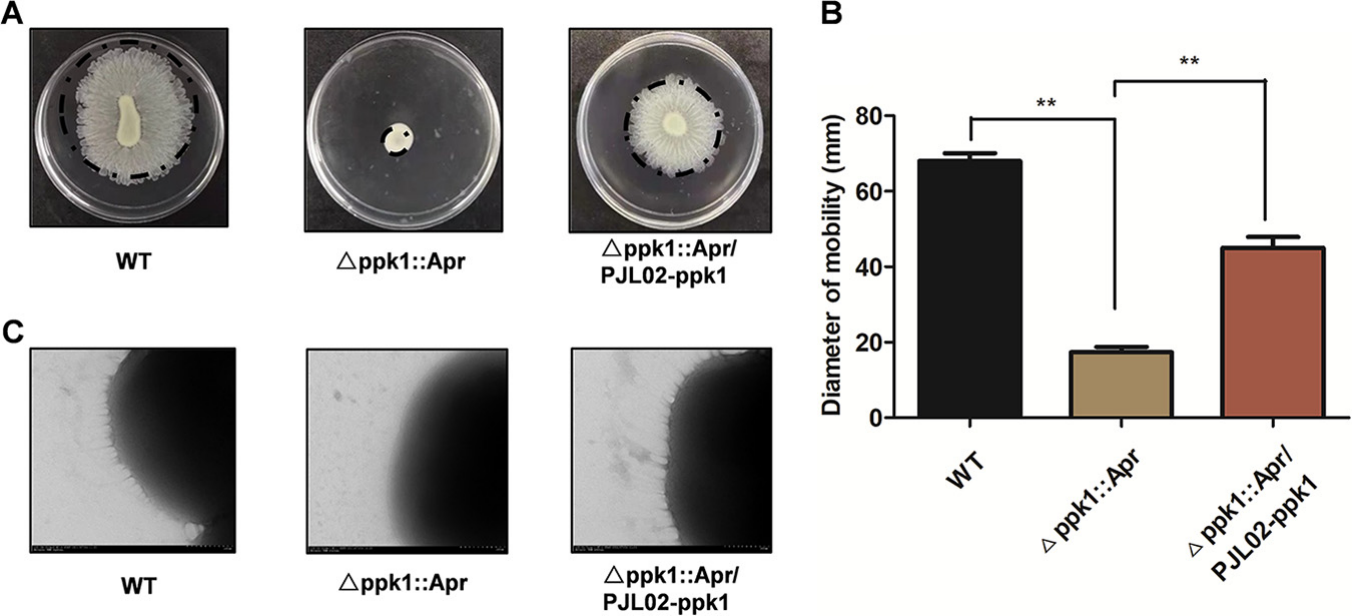
PPK1 is involved in surface-associated motility of A. baumannii. (A) Surface motility images of the WT and its derivative strains after incubation at 37°C with 5% CO_2_ for 14 h. (B) Surface motility diameter measurements from the images in panel A. (C) TEM images of the WT, Δ*ppk1*::Apr, and Δ*ppk1*::Apr/PJL02-*ppk1* strains in surface motility assay.

Surface motility assay results showed that the *ppk1-*deficient A. baumannii Δ*ppk1*::Apr strain lost its surface motility on plates compared with the WT or Δ*ppk1*::Apr/PJL02-*ppk1* strain (Fig. 2A). The average diameter of motility of the Δ*ppk1*::Apr strain (17 ± 2.51 mm) was significantly smaller than those of the WT (68 ± 3.60 mm) and Δ*ppk1*::Apr/PJL02-*ppk1* (45 ± 5.00 mm) strains (Fig. 2B). Transmission electron microscopy (TEM) was used to further determine whether the deficiency in PPK1 could affect the motility of A. baumannii. As shown in Fig. 2C, the Δ*ppk1*::Apr strain possessed a lost pilus-like structure compared with those of the WT and Δ*ppk1*::Apr/PJL02-*ppk1* strains. Thus, *ppk1* is required for the surface-associated motility of A. baumannii.

### PPK1 is vital for biofilm formation by A. baumannii.

**FIG 3:**
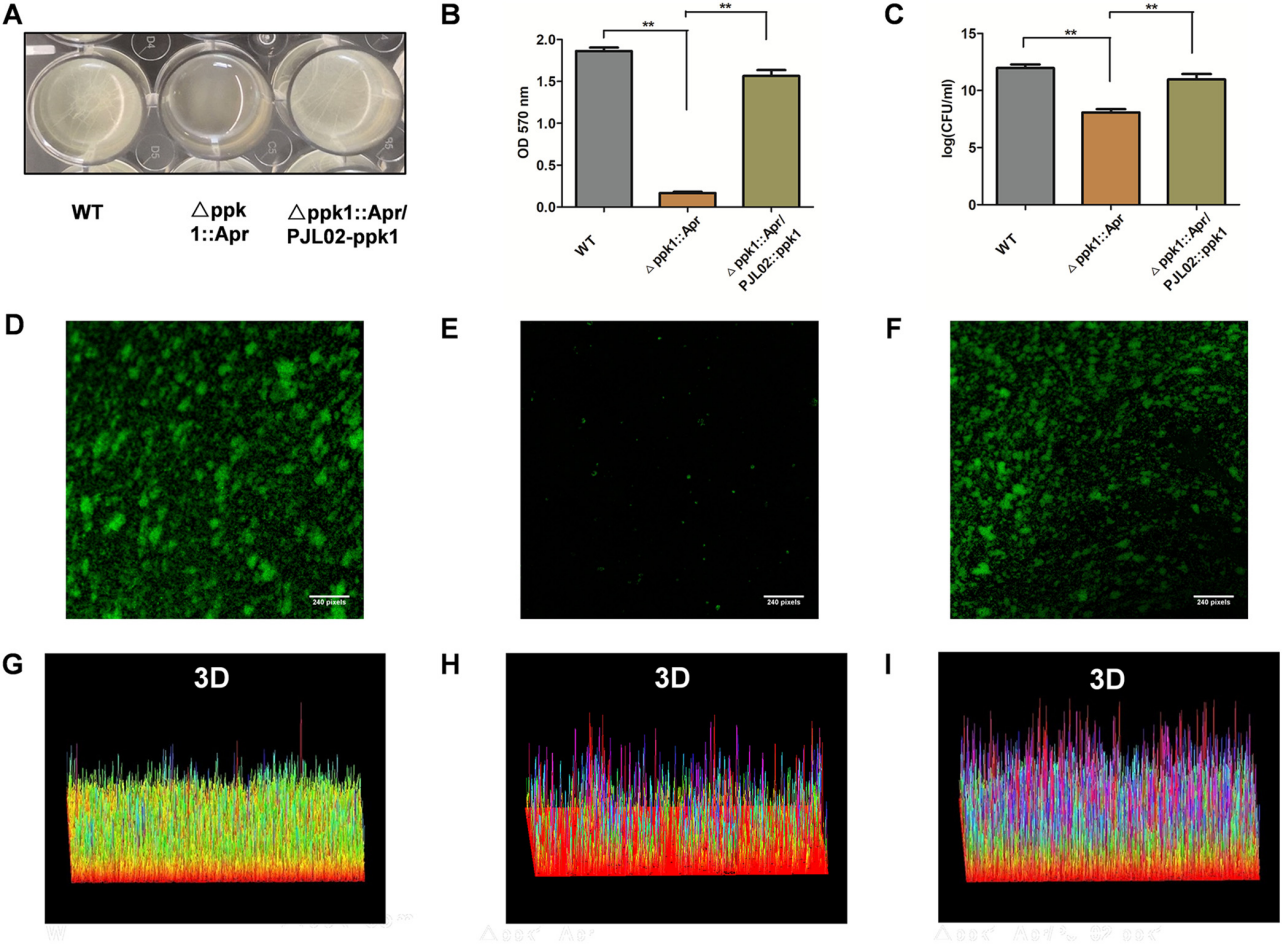
PPK1 is vital for biofilm formation by A. baumannii. (A) Overview of biofilm formation in the WT, Δ*ppk1*::Apr, and Δ*ppk1*::Apr/PJL02-*ppk1* strains after incubation at 30°C for 24 h. (B and C) Crystal violet assay and plate count method for biofilm quantitation of the WT and its derivative strains shown in panel A. (D to I) One-dimensional and three-dimensional images of biofilms of the WT, Δ*ppk1*::Apr, and Δ*ppk1*::Apr/PJL02-*ppk1* strains under a confocal microscope after analysis by ImageJ software.

In the biofilm formation assay, the WT and Δ*ppk1*::Apr/PJL02-*ppk1* strains showed a layer of pellicles on the surface of the liquid (Fig. 3A). However, no pellicle was observed in the Δ*ppk1*::Apr strain (Fig. 3A). A crystal violet assay was conducted to quantitate the biomass of the biofilm. The results indicated that the biofilm of the Δ*ppk1*::Apr strain was significantly reduced by 91.0% and 89.3% compared to those of the WT and Δ*ppk1*::Apr/PJL02-*ppk1* strains, respectively, as shown in Fig. 3B. In addition, biofilms can be quantitated by plate counting. As shown in Fig. 3C, the biofilm of the Δ*ppk1*::Apr strain was also reduced compared to that of the WT or Δ*ppk1*::Apr/PJL02-*ppk1* strain. As shown in Fig. 3D and G, the biofilm mass of the WT was similar to that of the Δ*ppk1*::Apr/PJL02-*ppk1* strain (Fig. 3F and I) under confocal laser scanning microscopy. Moreover, the Δ*ppk1*::Apr biofilm was much smaller, as shown in Fig. 3E and H. Images of the WT, Δ*ppk1*::Apr, and Δ*ppk1*::Apr/PJL02-*ppk1* strains were transformed into three-dimensional (3D) views using ImageJ software. From the 3D image, the thickness of the Δ*ppk1*::Apr biofilm was lower than those of the others (Fig. 3G to I). In conclusion, *ppk1* is essential for biofilm formation by A. baumannii.

### PPK1 is important for the persistence of A. baumannii.

**FIG 4:**
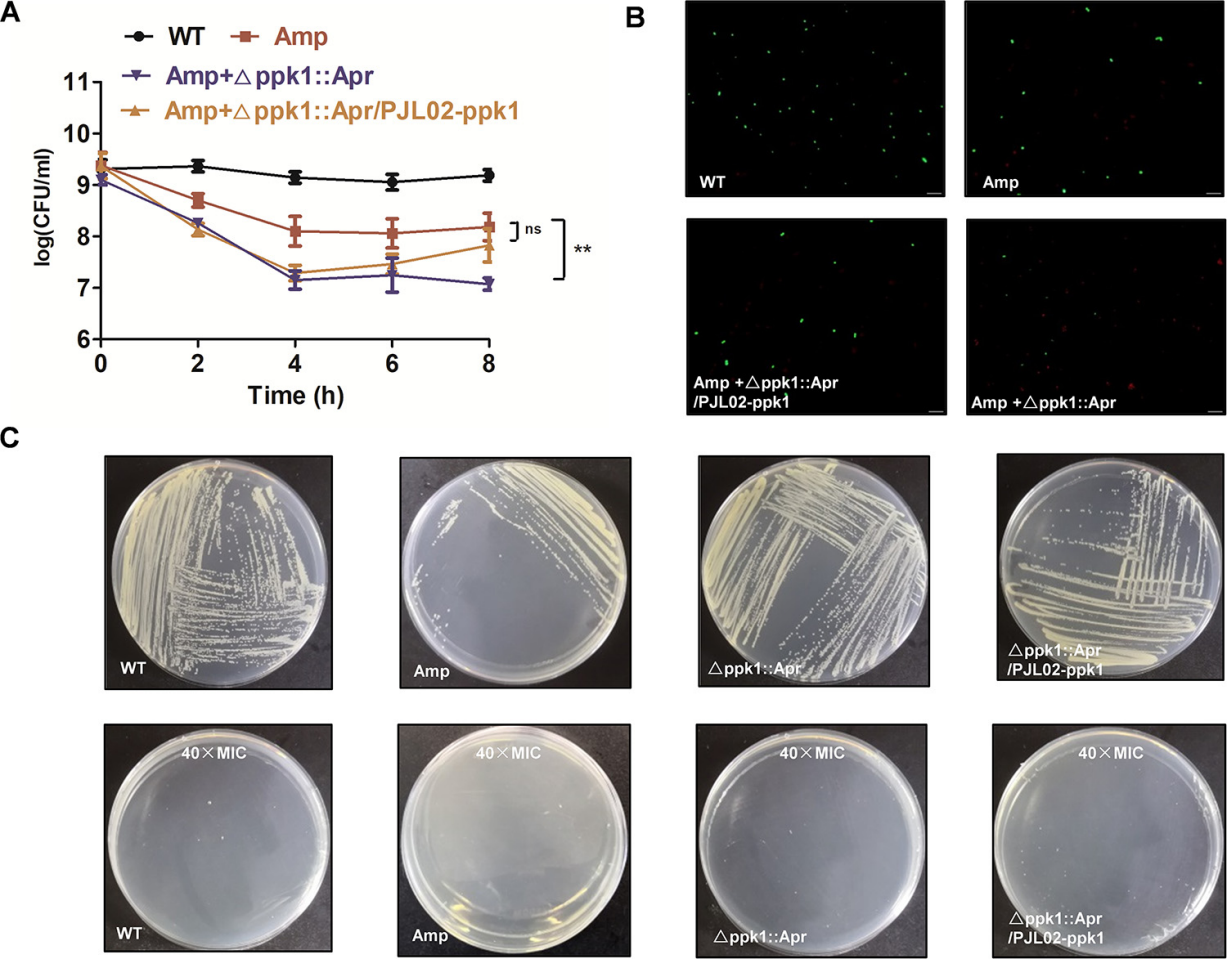
PPK1 is important for the persistence of A. baumannii. (A) Time-kill curves of the WT and it derives under treatment with 40× MIC of Amp for 8 h. (B) BacLight Live/Dead staining assay of the WT, Δ*ppk1*::Apr, and Δ*ppk1*::Apr/PJL02-*ppk1* strains with or without treatment with 40× MIC of Amp for 8 h. (C) Overview of A. baumannii persisters after subculture on LB agar plates with or without 40× MIC of Amp.

The persistence ability of A. baumannii is always associated with drug resistance. The results of MIC tests showed that the MICs of ampicillin (Amp) against the WT, Δ*ppk1*::Apr, and Δ*ppk1*::Apr/PJL02-*ppk1* strains were all 16 μg/mL. Sub-population bacteria called “persister” could be transformed into dormant state under high concentration of antibiotics to resist antibiotics stress. As shown in Fig. 4A, the results indicated that the “persister” population of the Δ*ppk1*::Apr strain was significantly smaller, about 1.1 log_10_ units, than that of the WT after Amp treatment at 40× MIC for 8 h. As expected, the Δ*ppk1*::Apr/PJL02-*ppk1* strain reversed the phenotypic change in the persistence ability of A. baumannii, whose persister population was 7.8 log_10_ units. Moreover, a BacLight Live/Dead staining assay was performed to visualize the persister population after Amp treatments at 40× MIC for 8 h. In the Amp treatments of the WT shown in Fig. 4B, the number of live bacteria that stained green was lower than that in the control group without Amp treatment. The results for the Δ*ppk1*::Apr/PJL02-*ppk1* group shown in Fig. 4B were similar to those for the Amp-treated WT group. However, many more dead bacteria that were dyed red were observed in the Δ*ppk1*::Apr group under Amp treatments, as shown in Fig. 4B. Persister formation is a phenotypic change that can tolerate high antibiotic doses (17). After the persister population was passaged on an agar plate, it could not grow on a 40× MIC Amp agar plate, as presented in Fig. 4C. These results illustrated that bacterial persistence could not be inherited. In summary, *ppk1* is important for the persistence of A. baumannii.

### PPK1 is necessary for A. baumannii to resist hydrogen, heat, and starvation stimuli.

**FIG 5:**
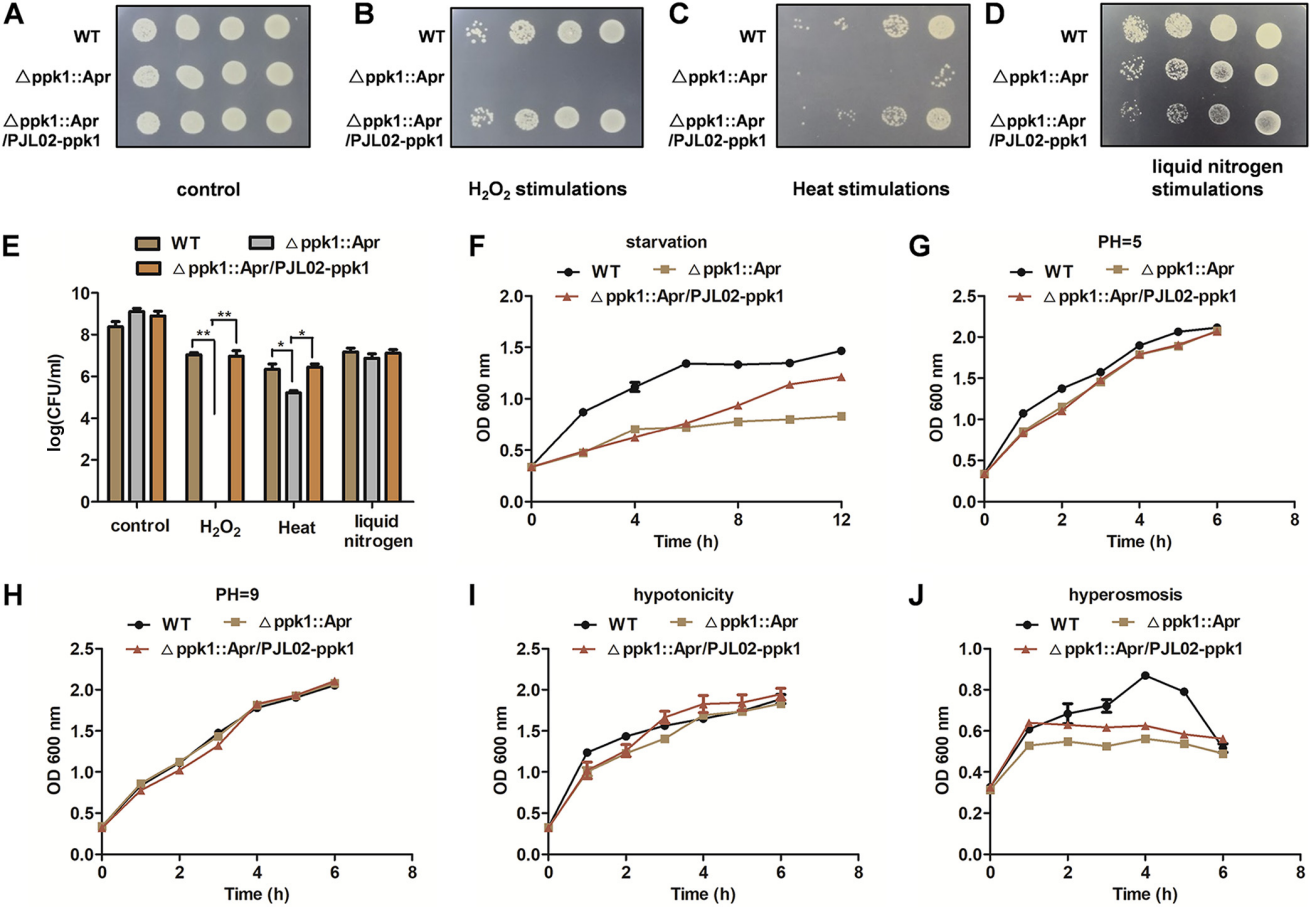
PPK1 is necessary for A. baumannii to resist hydrogen, heat, and starvation stimulation. (A to E) Overview and plate counts of bacterial survival before and after 0.05 mM hydrogen treatment for 30 min, heat stimulation at 60°C for 7 min, and liquid nitrogen stimulation for 30 s. (F) Growth curve determination for the WT, Δ*ppk1*::Apr, and Δ*ppk1*::Apr/PJL02-*ppk1* strains under starvation stimulation. (G to J) Growth curves of the WT, Δ*ppk1*::Apr, and Δ*ppk1*::Apr/PJL02-*ppk1* strains under acid (pH 5), base (pH 9), hypotonicity, and hyperosmotic stimulation.

To further explore the characteristics of *ppk1* against external adverse stimuli, including hydrogen peroxide, heat, liquid nitrogen, hyperosmosis, hypotonicity, starvation, acids, and bases, a stimulation assay was performed. As shown in Fig. 5A, similar growth states were observed for the WT, Δ*ppk1*::Apr, and Δ*ppk1*::Apr/PJL02-*ppk1* strains on agar without stimulation. The results of the hydrogen peroxide stimulus assay indicated that the deficiency of *ppk1* led to A. baumannii sensitivity to hydrogen peroxide, for which the amount of viable bacteria was decreased by 7.0 log_10_ units compared to that of the WT, as shown in Fig. 5B and E. The complemented Δ*ppk1*::Apr/PJL02-*ppk1* strain was observed to reverse these phenotypic changes. As shown in Fig. 5C, when the WT, Δ*ppk1*::Apr, and Δ*ppk1*::Apr/PJL02-*ppk1* strains were heated at 60°C for 7 min, the bacterial counts of the Δ*ppk1*::Apr strain were obviously reduced by 1.0 log_10_ units compared with those of the WT. However, the Δ*ppk1*::Apr strain was not sensitive to liquid nitrogen stimulation, as shown in Fig. 5D. In addition, the plate count method was performed to determine the number of viable cells before and after hydrogen peroxide, heat, and liquid nitrogen stimulation, as shown in Fig. 5E. Under starvation stimulation, the growth of the Δ*ppk1*::Apr strain was inhibited compared with those of the WT and Δ*ppk1*::Apr/PJL02-*ppk1* strains according to the growth curves shown in Fig. 5F. However, for the stimulation analyses with acids, bases, hyperosmosis, and hypotonicity, similar growth states of the WT and its derivatives were observed, as shown in Fig. 5G to J. Notably, the growth rate of the Δ*ppk1*::Apr strain was much lower than that of the WT with hyperosmotic stimulation at 4 h but not at 6 h (Fig. 5J). Taken together, our results indicated that *ppk1* is necessary for A. baumannii to resist hydrogen, heat, and starvation stimulation.

### PolyP can compensate for phenotypic changes of the Δ*ppk1*::Apr strain in bacterial persistence, starvation stimulation, and H_2_O_2_ stimulation assays.

**FIG 6:**
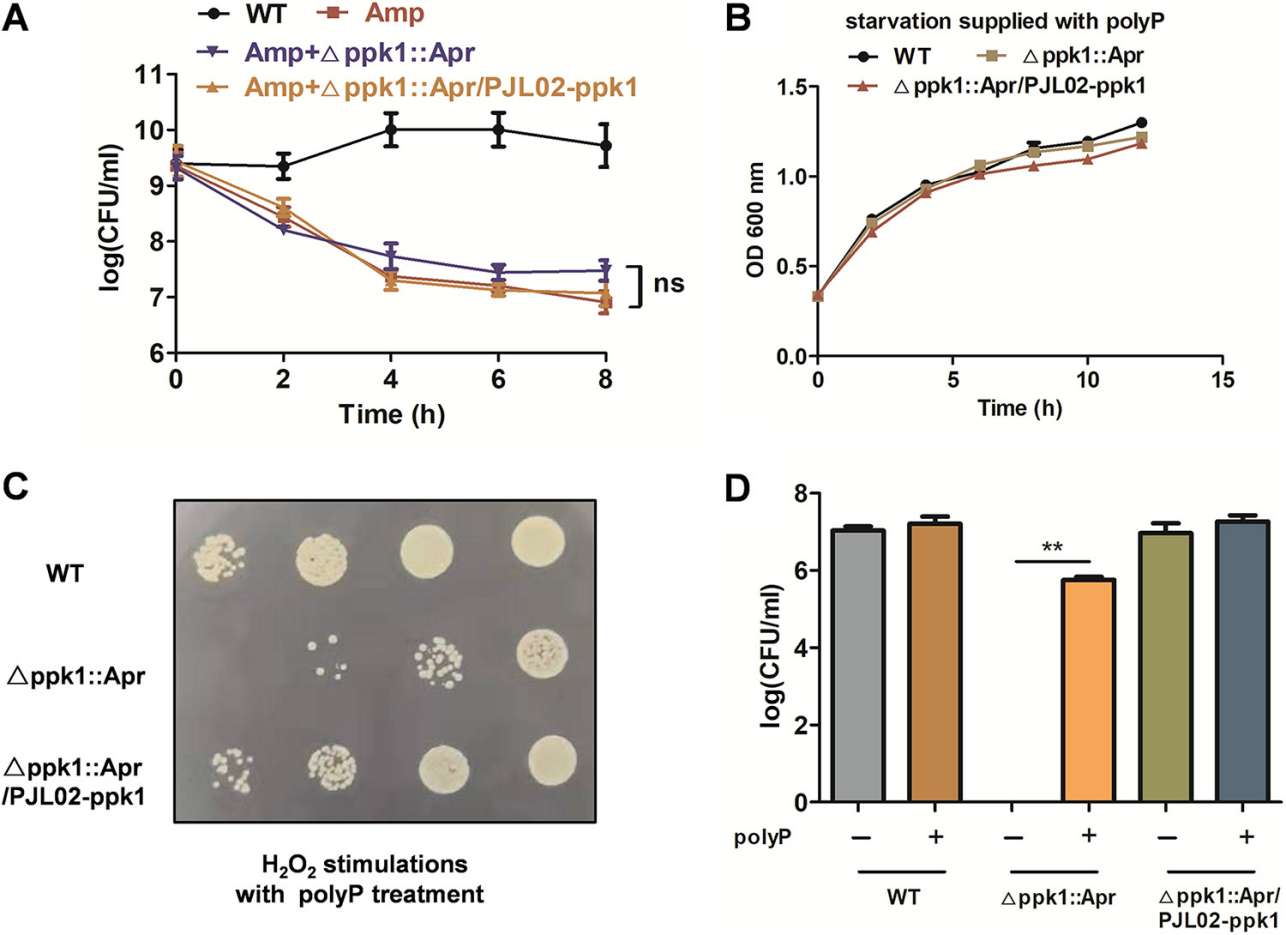
PolyP can compensate for the phenotypic changes of the Δ*ppk1*::Apr strain in bacterial persistence, starvation stimulation, and H_2_O_2_ stimulation assays. (A) The addition of 200 μg/mL polyP reversed the persistence of the Δ*ppk1*::Apr strain under treatment with 40× MIC of Amp for 8 h. (B) Growth curves of the WT, Δ*ppk1*::Apr, and Δ*ppk1*::Apr/PJL02-*ppk1* strains supplemented with 200 μg/mL polyP under starvation stimulation. (C and D) Overview and plate counts of bacterial survival after hydrogen peroxide treatment with 200 μg/mL polyP supplementation.

As shown in Fig. 6A, when polyP was cocultured with bacteria, the Δ*ppk1*::Apr strain was no different from the WT or Δ*ppk1*::Apr/PJL02-*ppk1* strain under Amp treatment. This result indicated that the addition of polyP could reverse this phenotypic change of the Δ*ppk1*::Apr strain. The average persister populations were 6.9 log_10_ units, 7.4 log_10_ units, and 7.0 log_10_ units for the WT, Δ*ppk1*::Apr, and Δ*ppk1*::Apr/PJL02-*ppk1* strains, respectively (Fig. 6A). In starvation assays, when WT, Δ*ppk1*::Apr, and Δ*ppk1*::Apr/PJL02-*ppk1* cultures were supplied with 200 μg/mL polyP, the Δ*ppk1*::Apr strain could resume growing, as shown in Fig. 6B. Next, hydrogen peroxide was also applied to explore whether the addition of polyP could reverse these phenotypic changes in the Δ*ppk1*::Apr strain. The results (Fig. 6C) indicated that Δ*ppk1*::Apr strain viability was recovered, whose amount of viable bacteria was 5.7 og_10_ units (Fig. 6D) compared with no polyP additions (Fig. 5B). The plate count method and statistical significance analysis are also shown in Fig. 6D.

### PPK1 is associated with glycerophospholipid metabolism and fatty acid biosynthesis in A. baumannii.

**FIG 7:**
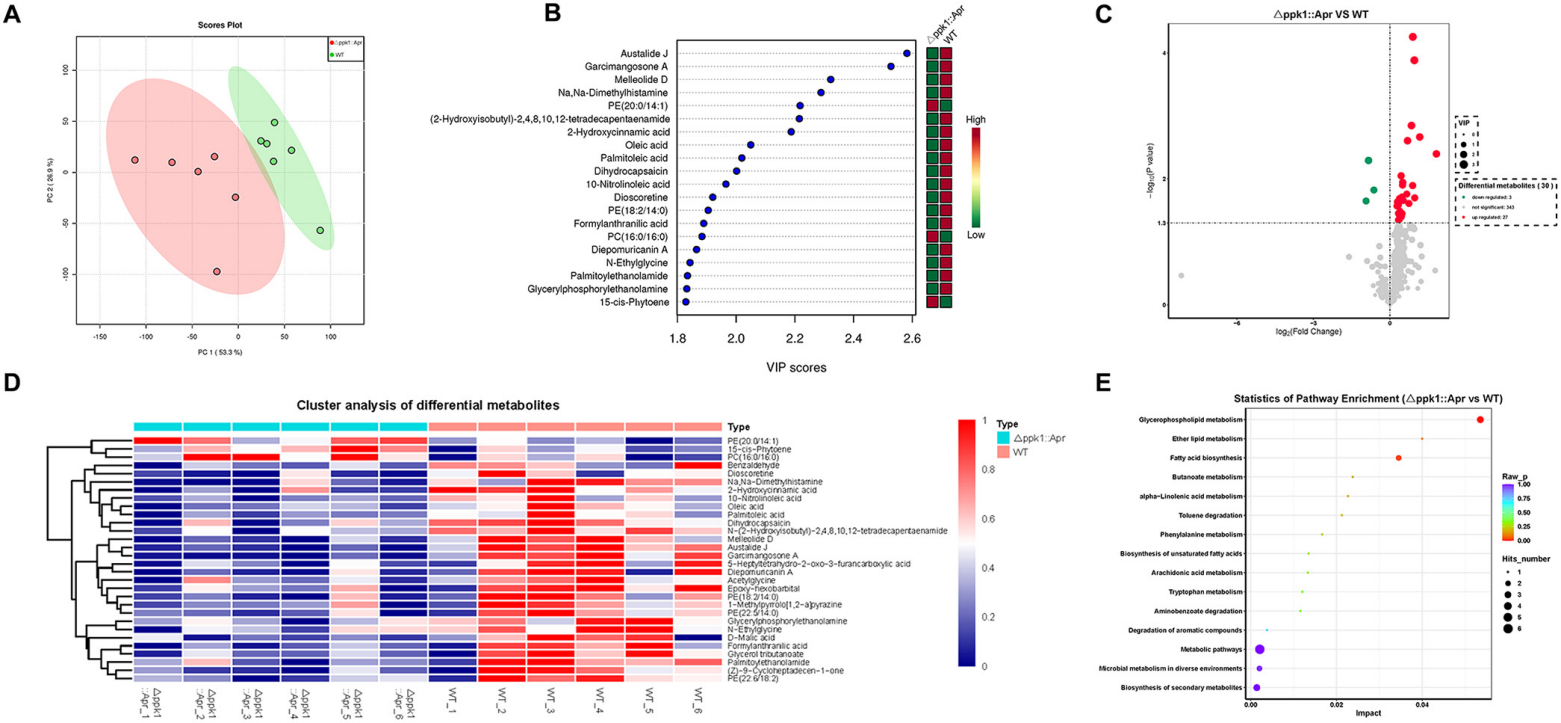
PPK1 is associated with glycerophospholipid metabolism and fatty acid biosynthesis in A. baumannii. (A) Score plots of metabolic variation principal-component analysis of the WT and Δ*ppk1*::Apr strains. (B) OPLS-DA of the WT and Δ*ppk1*::Apr strains. (C) Student’s *t* test of the differential metabolites between the WT and Δ*ppk1*::Apr strains. (D) Cluster analysis of the differential metabolites between the WT and Δ*ppk1*::Apr strains. (E) Differential metabolite pathways between the WT and Δ*ppk1*::Apr strains by KEGG analysis. PE, phosphatidylethanolamine; PC, phosphatidylcholine.

A. baumannii metabolomics analysis was performed using an ultrahigh-performance liquid chromatography (UHPLC) system (Vanquish; Thermo Fisher Scientific, USA) and a Q Exactive HFX mass spectrometer (Orbitrap MS; Thermo) in information-dependent acquisition (IDA) mode. As presented in Fig. 7A, metabolic variation analysis showed that the score plots for the WT and Δ*ppk1*::Apr groups clustered well and separated clearly. Significant metabolites were selected based on a variable importance in the projection (VIP) value of >1.0 and a *P* value of <0.05 using orthogonal partial least-squares discriminant analysis (OPLS-DA) and Student’s *t* test. Twenty-seven differentially downregulated metabolites and 3 upregulated metabolites were observed in the metabolomic analysis between the WT and Δ*ppk1*::Apr strains (Fig. 7B and C). In addition, hierarchical clustering was performed for these differential metabolites, as shown in the heatmap in Fig. 7D. Upregulated metabolites are marked in red, and downregulated metabolites are marked in blue in the heatmap. To determine the metabolic pathways associated with significant metabolites, the metabolic pathways related to the significant metabolites were determined by KEGG analysis. As shown in Fig. 7E, the significantly different metabolic pathways between the WT and Δ*ppk1*::Apr strains were glycerophospholipid metabolism and fatty acid biosynthesis. Therefore, our results suggested that *ppk1* was involved in these selected pathways.

### PPK1 enhanced A. baumannii survival and virulence in mice.

**FIG 8:**
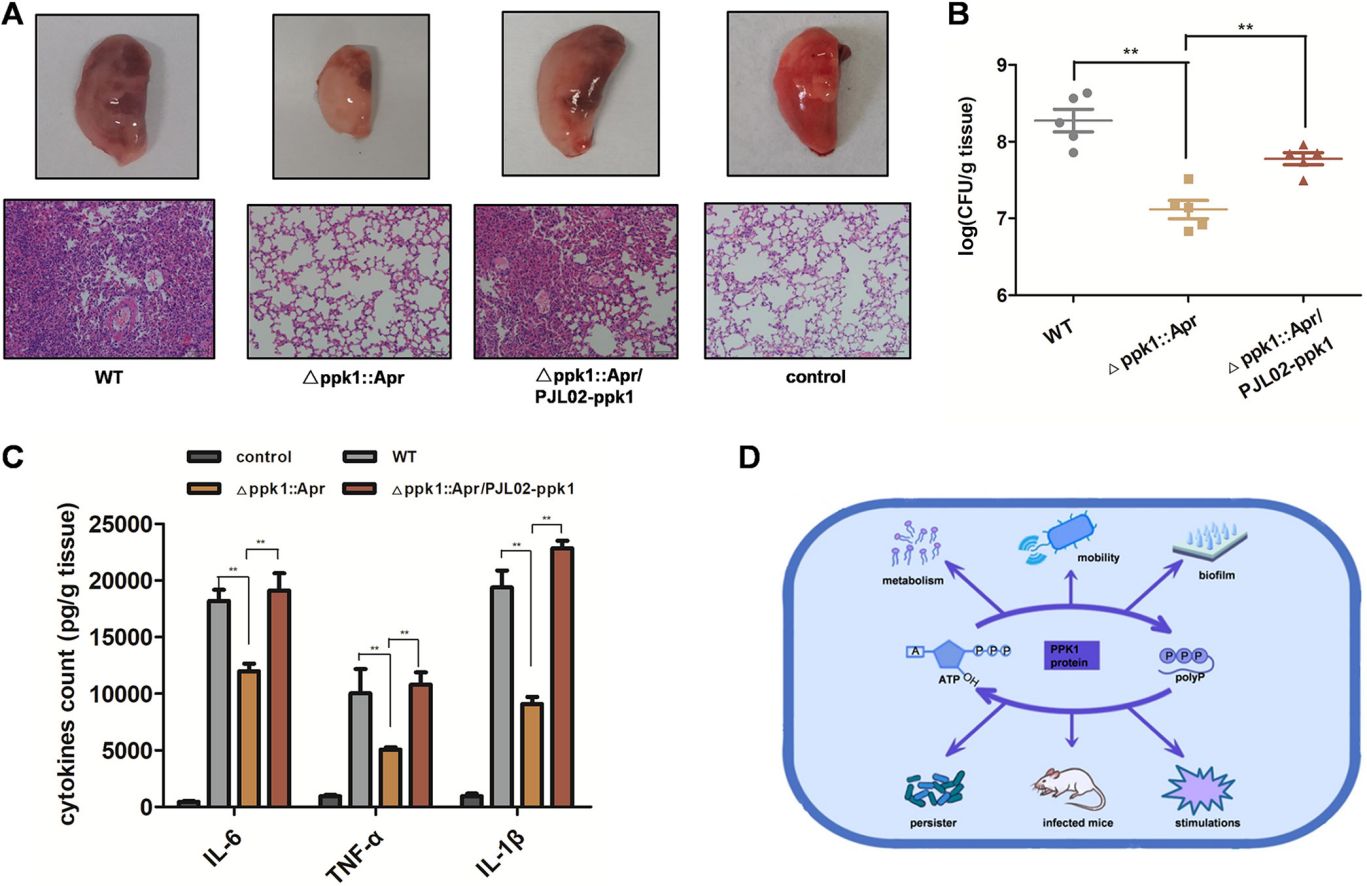
PPK1 enhanced A. baumannii survival and virulence in mice. (A) Gross lesion observation and H&E analysis of lungs of mice infected with the WT, Δ*ppk1*::Apr, or Δ*ppk1*::Apr/PJL02-*ppk1* strain. (B) Bacterial load determinations for the WT and its derivatives in infected lungs at 48 h postinfection. (C) IL-1β, IL-6, and TNF-α level determinations in lungs following WT, Δ*ppk1*::Apr, and Δ*ppk1*::Apr/PJL02-*ppk1* infection for 48 h. (D) Schematic summary of the role of PPK1 in A. baumannii.

A mouse pneumonia model was established by nasal perfusion to explore the role of PPK1 in the mammalian host. BALB/c mice were infected with the WT, Δ*ppk1*::Apr, and Δ*ppk1*::Apr/PJL02-*ppk1* strains separately. After infection for 48 h, significant pneumonia injury, including pulmonary internal hemorrhage, blood clot, and edema, were observed in WT-infected mice (Fig. 8A). However, these pneumonia injuries were much relieved in Δ*ppk1*::Apr-infected mice compared to those in WT-infected mice. Pulmonary internal hemorrhage, blood clot, edema, and inflammatory changes were also observed in the Δ*ppk1*::Apr/PJL02-*ppk1* group (Fig. 8A). In agreement with the *in vitro* analysis, the Δ*ppk1*::Apr/PJL02-*ppk1* complementation strain restored the virulence of the Δ*ppk1*::Apr strain (Fig. 8A). In addition, the lungs of controls without bacterial infection appeared pale pink and had a normal anatomical form. Lung histological changes were also observed by hematoxylin and eosin (H&E) staining. As shown in Fig. 8A, the alveolar structure of the lung of the WT-infected mice was filled with neutrophil cells and bronchial epithelium shedding along with incomplete alveolar structures. However, such injuries were not observed in the mice that received the Δ*ppk1*::Apr strain or in uninfected mice. The H&E staining results for the Δ*ppk1*::Apr/PJL02-*ppk1* strain were also consistent with the results of gross lesion observation analysis.

Another mouse lung after WT, Δ*ppk1*::Apr, and Δ*ppk1*::Apr/PJL02-*ppk1* infection was homogenized and used for bacterial load determination. The bacterial loads of the WT and Δ*ppk1*::Apr/PJL02-*ppk1* strains were approximately 6.89 ± 0.32 log_10_ units and 6.48 ± 0.17 log_10_ units, respectively (Fig. 8B). As expected, the bacterial load of the Δ*ppk1*::Apr strain was 5.92 ± 0.26 log_10_ units, which was approximately 1 log_10_ unit lower than those of the WT and Δ*ppk1*::Apr/PJL02-*ppk1* strains (Fig. 8B). Thus, PPK1 is required for A. baumannii-mediated tissue injury. In addition, an enzyme-linked immunosorbent assay (ELISA) was conducted to determine the levels of cytokines (interleukin-1β [IL-1β], IL-6, and tumor necrosis factor alpha [TNF-α]) in the lungs of mice. The results indicated that the IL-1β, IL-6, and TNF-α levels in Δ*ppk1*::Apr-infected mice were significantly reduced compared with those in WT- or *ppk1*::Apr/PJL02-*ppk1*-infected mice (Fig. 8C). Together, our results established that PPK1 could enhance A. baumannii survival and virulence in mice.

## DISCUSSION

A. baumannii is a multidrug-resistant bacterium that can tolerate a variety of adverse stimuli such as desiccation, heat, and disinfectants (1). PPK, as a polyP synthase, is associated with bacterial virulence processes, such as biofilm formation and motility, including bacterial persistence (18, 19). However, whether PPK affects the virulence and persistence of A. baumannii is not well characterized. To the best of our knowledge, this is the first study to generate *ppk1*-deficient and *ppk1*-complemented strains of A. baumannii ATCC 17978 to explore the role of PPK in A. baumannii virulence and persistence in our study.

Our results showed that PPK1 was essential for polyP accumulation *in vivo* and associated with ATP metabolism. These observed PPK biological functions were consistent with those for Mycobacterium tuberculosis and Pseudomonas aeruginosa (20, 21). The metabolites 10-nitrolinoleic acid, glycerylphosphorylethanolamine, and oleic acid, which belong to the glycerophospholipid metabolism and fatty acid biosynthesis pathways, were downregulated in the Δ*ppk1*::Apr strain (Fig. 7B). Previous work showed that various polar head groups of glycerophospholipids could be attached to the surface of phosphatidic acid in bacteria to supply the preferred membrane surface charge (22), which facilitates bacterial adaption to various external stress conditions (23). Thus, the downregulation of glycerophospholipid metabolism in the Δ*ppk1*::Apr strain may alter the membrane charge, resulting in an adverseness of bacteria to heat, hydrogen peroxide stress, or high-concentration antibiotic pressure. Additionally, polyP accumulation in bacteria was associated with glycerol 3-phosphate, which is required for the generation of phospholipids involved in glycerophospholipid metabolism, glycerol, and polyP (13, 24). Therefore, polyP is closely related to glycerophospholipid metabolism and bacterial resistance abilities in bacteria.

In line with these observations, we also found that deficient strains exhibited impairments in motility (Fig. 2A) and biofilm formation (Fig. 3A). The role of PPK1 in A. baumannii persistence was consistent with previous reports on other bacteria (12, 13, 24), and the persistence ability of *ppk1*-deficient strains was reduced significantly compared with that of the WT strain under Amp treatment (Fig. 4A). Similar to antibiotic persistence, the results of hydrogen peroxide, heat, liquid nitrogen, hyperosmosis, hypotonicity, starvation, acid, and base stimulation assays indicated that the Δ*ppk1*::Apr strain was sensitive to hydrogen peroxide, heat, and starvation stimulation (Fig. 5B, C, and F), which was consistent with the role of PPK in other bacteria (12, 25). However, in other stimulation assays, PPK1 did not affect the sensitivity of A. baumannii (Fig. 5G to J).

PolyP, as a primordial chaperone, could prevent protein aggregation under hydrogen peroxide and heat treatment (7). In this work, the addition of polyP could reverse this phenotypic change of the Δ*ppk1*::Apr strain in a hydrogen peroxide stimulation assay (Fig. 6C). Interestingly, additions of polyP also reversed the phenotypic changes in antibiotic persistence and starvation stress (Fig. 6A and B). PolyP has been shown to combine with Lon to degrade antitoxins and induce persistence (17). For the starvation stimulation assay, the added polyP might serve as an energy source to compensate for the growth inhibition of the Δ*ppk1*::Apr strain (26). Furthermore, PPK1 was essential for A. baumannii virulence and persistence *in vitro* and *in vivo*. However, the mechanism of PPK1 for such regulation must be further determined.

Our work indicated that PPK1 had significant effects on virulence, antibiotic persistence, stress tolerance, and pathogenesis (Fig. 8D). In addition, PPKs are conserved in many bacteria and deficient in mammals. Thus, PPKs could be a new target for combating A. baumannii persistence and virulence, with further work focusing on the identification of PPK inhibitors.

## MATERIALS AND METHODS

### Bacterial strains, reagents, plasmids, and culture conditions.

4′,6-Diamidino-2-phenylindole (DAPI) and FLAG tag antibody were purchased from Sigma (St. Louis, MO, USA). Cytokine detection kits and BacLight Live/Dead staining kits were purchased from Invitrogen (Carlsbad, CA, USA). Polyphosphate (polyP) was purchased from Aladdin (Shanghai, China). Morpholinepropanesulfonic acid (MOPS) minimal medium was purchased from Teknova. The plasmids and bacterial strains used in this study are listed in Table S1 in the supplemental material.

### Generation of *ppk1* knockout and *ppk1*-complemented strains from A. baumannii ATCC 17978.

The *ppk1*-deficient strain of A. baumannii ATCC 17978 (Δ*ppk1*::Apr) was constructed according to previously reported methods (27). The primers for the amplification of the open reading frame of *ppk1* (GenBank accession number CP053098.1), upstream and downstream homologous recombination DNA fragments, and the apramycin resistance gene from plasmid pUC57-Apr are listed in Table S2. In brief, *ppk1* was replaced by an apramycin resistance gene through a fusion PCR technique and cloned into plasmid pCVD442 using restriction enzymes and T4 DNA ligases. Next, the plasmid was transformed into E. coli β2155 to combine with A. baumannii ATCC 17978. 300 μg/mL apramycin-containing LB agar plates were coated with the products of conjunction, and the products were selected by PCR using primer pairs *ppk1*-out forward/reverse and *Apr*-seq forward/reverse. Furthermore, after sucrose-LB agar plate screening, the correct clone was identified by PCR using the primers (*ppk1*-inF/*ppk1*-inR and *ppk1*-outF/*ppk1*-outR) listed in Table S2. To generate the *ppk1-*complemented strain, *ppk1* was amplified from A. baumannii using primers *ppk1*-BamHI-F and *ppk1*-SalI-R and cloned into plasmid PJL02 (28). The constructed plasmid, PJL02-*ppk1*, was transformed into the donor strain E. coli WM3064, which was further combined with the Δ*ppk1*::Apr strain. The correct clone was identified by PCR using the primer pair PJL02-outF2/R.

### Western blot assay.

The Δ*ppk1*::Apr/PJL02-*ppk1* strain grown overnight was diluted into fresh LB broth containing 0 mM, 0.25 mM, 0.5 mM, 1 mM, or 2 mM isopropyl-β-d-thiogalactopyranoside (IPTG) with shaking (180 rpm at 37°C). When the strain reached the logarithmic phase, the bacterial precipitate was collected at 12,000 rpm for Western blotting with 12% sodium dodecyl sulfate-polyacrylamide gel electrophoresis. The polyvinylidene difluoride (PVDF) membrane was blocked with 5% fat-free milk and incubated with FLAG tag antibody (Sigma, USA) for 1 h at room temperature. After washing with Tris-buffered saline–Tween (TBST) three times, the PVDF membrane was placed into a Tanon‐4200 imager for imaging to analyze the expression level of PPK1.

### Growth curve determination.

To determine whether the deficiency of PPK1 influences the growth status of A. baumannii under LB culture conditions, the growth trend was examined according to methods in a previous report (29). Cultures of the WT, Δ*ppk1*::Apr, and Δ*ppk1*::Apr/PJL02-*ppk1* A. baumannii strains grown overnight were diluted 100-fold into 20 mL LB medium with shaking at (180 rpm at 37°C). The growth curve of A. baumannii was monitored every hour once the optical density at 600 nm (OD_600_) reached 0.3. In addition, the effect of polyP on bacterial growth was also determined.

### Quantification of polyP in A. baumannii under nutrient deprivation.

A polyP quantification assay was performed to explore whether PPK1 deficiency has an effect on bacterial polyP accumulation (30). Briefly, cultures of A. baumannii grown overnight were diluted into fresh LB broth and cultured (180 rpm at 37°C) to mid-log phase. The bacterial pellet was resuspended in MOPS low-phosphate minimal medium (0.2% glucose, 100 μM potassium phosphate, and 10 μM thiamine) and incubated at 37°C in a 5% CO_2_ incubator for 2 h to stimulate the accumulation of polyP. Next, 1 mL of bacterial cells (OD_600_ = 0.6) was centrifuged at 10,000 rpm at 4°C for 5 min. Bacterial cells were further suspended in 50 mM HEPES buffer (pH 7.5) and heated at 60°C for 10 min to increase bacterial membrane permeability. Next, the pellet was resuspended in DAPI assay buffer (150 mM KCl, 20 mM HEPES-KOH, 10 μM DAPI [pH 7.0]) and incubated for 10 min at room temperature in the dark. The fluorescence intensity of the DAPI-polyP complex in A. baumannii was determined with excitation at 415 nm and emission at 550 nm. To visualize the polyP localization and quantity in bacteria (16), the DAPI-PolyP complex was also observed under a confocal microscope (Olympus).

### Measurement of intracellular ATP levels.

The synthesis of polyP is catalyzed by PPK from ATP under nutrient deprivation (15). Therefore, the intracellular ATP level could reflect the biological function of PPK. Briefly, cultures of the WT, Δ*ppk1*::Apr, and Δ*ppk1*::Apr/PJL02-*ppk1* strains grown overnight were expanded into LB medium with shaking at 37°C for 2.5 h, and logarithmic-phase bacterial cells were then collected at 12,000 rpm at 4°C after nutrient deprivation with MOPS low-phosphate minimal medium for 2 h. The intracellular ATP level of A. baumannii was determined by an ATP detection kit (Beyotime) using a microplate reader (Synergy H1; BioTek).

### Surface motility of A. baumannii.

A surface motility assay of A. baumannii was performed according to previously published protocols, with some modifications (31, 32). Cultures of the WT, Δ*ppk1*::Apr, and Δ*ppk1*::Apr/PJL02-*ppk1* strains grown overnight were diluted to an OD_600_ value of 0.5 with LB broth. Next, 2 μL of the bacterial suspension was dropped on the middle of agar plates (10 g/L tryptone, 5 g/L yeast extract, 1 g/L glucose, 5 g/L NaCl, and 3 g/L agar). After incubation at 37°C for 14 h, photographs of A. baumannii on plates were obtained, and the diameter of motility was measured.

### Transmission electron microscopy assay.

A transmission electron microscopy (TEM) assay was used to explore the differences in motility among the WT, Δ*ppk1*::Apr, and Δ*ppk1*::Apr/PJL02-*ppk1* strains of A. baumannii ATCC 17978 (31). Colonies of the WT, Δ*ppk1*::Apr, and Δ*ppk1*::Apr/PJL02-*ppk1* strains were resuspended in 100 μL phosphate-buffered saline (PBS), from which a 10-μL sample was spilt on standard copper TEM grids and incubated for 5 min. Bacterial samples were stained with uranyl acetate and observed using a transmission electron microscope (Hitachi).

### Biofilm assay and quantification.

A biofilm assay was conducted according to previously described guidelines (33, 34). Briefly, 2 × 10^5^ CFU/mL bacterial cells of the WT, Δ*ppk1*::Apr, and Δ*ppk1*::Apr/PJL02-*ppk1* strains of A. baumannii ATCC 17978 were applied to each well (24-well plates) with Mueller-Hinton broth (MHB) and incubated statically (30°C with 5% CO_2_) for 24 h. The wells were washed twice with PBS to remove planktonic cells after being photographed. The biofilm was dried at 37°C for 30 min and stained with 1 g/L crystal violet at room temperature for 1 h. Excess crystal violet was washed away with PBS two times. Next, the biofilms were solubilized with 33% acetic acid and quantified by determining the absorbance at 570 nm. Bacterial loading in biofilm-attached wells was also confirmed by a 10-fold serial dilution method in LB agar plates. After incubation at 37°C for 12 h, the colonies on the agar plates were counted and analyzed.

### Confocal laser scanning microscopy.

The biofilms of the WT, Δ*ppk1*::Apr, and Δ*ppk1*::Apr/PJL02-*ppk1* strains of A. baumannii ATCC 17978 were further observed using SYTO-9–propidium iodide (PI) by confocal laser scanning microscopy (35). A. baumannii (2 × 10^5^ CFU/mL) was cultured in MHB at 30°C for 24 h in a 24-well plate with slides. After incubation, the wells were washed twice with PBS. Biofilms were stained with a BacLight Live/Dead staining kit (Invitrogen) in the dark for 15 min according to the manufacturer’s instructions, observed under a confocal laser scanning microscope (Olympus), and analyzed using ImageJ software.

### MIC determination.

MIC assays for the WT, Δ*ppk1*::Apr, and Δ*ppk1*::Apr/PJL02-*ppk1* strains were performed by a method of serial 2-fold dilution, essentially as described in the Clinical and Laboratory Standards Institute (CLSI) protocol (36). Briefly, 2-fold dilutions of Amp (0 μg/mL and 4 μg/mL to 256 μg/mL) were diluted with LB medium in the wells of the same row of a 96-well plate, and the tested bacteria were then grown for 18 to 24 h at 37°C in an incubator. The lowest concentrations of Amp resulting in clear cultures were visually determined as the MIC values of Amp.

### Bacterial persistence assay.

In order to determine the role of PPK1 under treatment with high concentrations of Amp, a bacterial persistence assay was performed as described in a previous study (18). Cultures of the WT, Δ*ppk1*::Apr, and Δ*ppk1*::Apr/PJL02-*ppk1* strains of A. baumannii ATCC 17978 grown overnight were incubated in fresh LB medium (1:100) at 37°C at 180 rpm for 2.5 h (OD_600_ = 0.6). Next, 40× MIC (640 μg/mL) of Amp was added to the cultures. Two milliliters of the bacterial suspension was collected and resuspended in PBS every 2 h. The total viable bacteria were serially diluted 10-fold and spread onto LB agar plates. The colonies on the agar plates were counted and analyzed after incubation at 37°C for 12 h.

To verify whether persisters could restore sensitivity to antibiotics in the absence of high-dose antibiotic pressure (19), the persistent bacteria were inoculated onto LB agar plates with or without 40× MIC (640 μg/mL) of Amp to observe the growth state.

### BacLight Live/Dead staining assay.

A BacLight Live/Dead staining assay was performed to further evaluate the bacterial retention states after treatment with 40× MIC (640 μg/mL) of Amp for 8 h (37). The bacteria were collected at 12,000 rpm at 4°C for 5 min and washed twice with PBS. The optical density at 600 nm was adjusted to 0.5 with PBS. BacLight Live/Dead staining reagent was added according to the manufacturer’s instructions. After incubation at room temperature for 15 min in the dark, 5 μL of the sample was dropped onto the slides and visualized under a confocal laser scanning microscope (Olympus).

### Survival assays of A. baumannii with stimulation under adverse conditions.

Cultures of the WT, Δ*ppk1*::Apr, and Δ*ppk1*::Apr/PJL02-*ppk1* strains of A. baumannii ATCC 17978 grown overnight were inoculated into fresh LB broth at a ratio of 1:100. Next, 1-mL logarithmic-phase bacterial pellets were collected and resuspended with an equivalent volume of PBS containing 0.05 mM H_2_O_2_ at room temperature for 30 min without shaking. A 100-μL sample was serially diluted 10-fold and spread onto an LB agar plate to determine bacterial survival. For the heat shock assay (24), 1-mL logarithmic-phase bacterial samples of the WT, Δ*ppk1*::Apr, and Δ*ppk1*::Apr/PJL02-*ppk1* strains were collected, suspended with an equivalent volume of PBS, and treated in a 60°C water bath for 7 min. Next, 100-μL samples were serially diluted 10-fold and spread onto LB agar to determine bacterial survival. For liquid nitrogen stimulation (38), 1 mL of logarithmic-phase bacteria was collected, frozen in liquid nitrogen for 30 s, and then thawed at 37°C in a 5% CO_2_ incubator. The survival of bacteria was also determined by the plate count method. For the starvation stress assay (39), bacteria cultured overnight were inoculated into fresh LB broth at a ratio of 1:100 to an OD_600_ of 0.3, and bacterial precipitates were transferred to MOPS minimal medium (0.2% glucose, 1.32 mM potassium phosphate, and 10 μM thiamine). The growth state of bacteria was monitored every hour. Other stressors, including acid or base stress and osmotic stress, were also explored in the WT, Δ*ppk1*::Apr, and Δ*ppk1*::Apr/PJL02-*ppk1* strains of A. baumannii ATCC 17978 (39). Normal LB broth was replaced by acidic LB broth (pH 5), basic LB broth (pH 9), hyperosmotic LB broth (10 g/L tryptone, 5 g/L yeast extract, 100 g/L NaCl), or hypotonic LB broth (10 g/L tryptone and 5 g/L yeast extract).

### Δ*ppk1*::Apr phenotype compensation assay.

PPK1 as a polyP synthase could affect the metabolism of polyP in bacteria. In our study, we sought to explore whether the addition of polyP could compensate for the phenotypic changes of the Δ*ppk1*::Apr strain. In a bacterial persistence assay, cultures of the WT, Δ*ppk1*::Apr, and Δ*ppk1*::Apr/PJL02-*ppk1* strains grown overnight were inoculated into fresh LB broth at a ratio of 1:100 with 200 μg/mL polyP for 2.5 h (OD_600_ = 0.6). Next, 40× MIC (640 μg/mL) of Amp was added to the cultures. The persisters were also determined by the plate counting method for 8 h.

For the starvation stress assay, the WT and its derived strains were inoculated into fresh LB broth to an OD_600_ of 0.3, and bacterial precipitates were transferred to MOPS minimal medium with shaking (180 rpm at 37°C) in the presence of 200 μg/mL polyP. The growth state of bacteria was also monitored every hour.

For the H_2_O_2_ stimulation assay, logarithmic-phase bacteria (OD_600_ = 0.6) cocultured with 200 μg/mL polyP were collected and resuspended in PBS containing 0.05 mM H_2_O_2_ at room temperature for 30 min. The plate counting method was performed to determine the survival of bacteria.

### Metabonomic analysis.

The WT and Δ*ppk1*::Apr strains of A. baumannii ATCC 17978 were cultured in LB broth and collected during logarithmic growth with the optical density at 600 nm adjusted to 0.1. Bacterial pretreatment methods were performed according to previous reports, with some modifications (40, 41). First, a 500-μL extract solution (acetonitrile-methanol-water, 2:2:1) containing an isotopically labeled internal standard mixture was added to the WT and Δ*ppk1*::Apr strains. After vortexing, the samples were frozen and thawed with liquid nitrogen 3 times. Next, the samples were sonicated for 10 min in an ice water bath. After the samples were incubated at −40°C for 1 h, they were centrifuged at 12,000 rpm for 15 min at 4°C, and the supernatant was collected and filtered through a 0.22-μm filter membrane before liquid chromatography-tandem mass spectrometry (LC-MS/MS) analysis.

Bacterial metabolites in the supernatants were determined by a UHPLC system (Vanquish; Thermo Fisher Scientific, USA) and a Q Exactive HFX mass spectrometer (Orbitrap MS; Thermo) in information-dependent acquisition (IDA) mode (42). Ammonium acetate (25 mM), ammonia hydroxide (pH 9.75), and acetonitrile were used as the mobile phases. The raw data were converted to mzXML format using ProteoWizard and processed with an in-house program, which was illustrated using the R software platform with XCMS (Xcmsonline.scripps.edu) for peak detection, extraction, alignment, and integration. Next, an in-house MS2 (mass spectrometric 2) database was applied for metabolite annotation. The cutoff for annotation was set at 0.3.

### A. baumannii*-*infected mouse pneumonia model.

Six- to eight-week-old female BALB/c mice (Liao Ning Chang Sheng Biotechnology Co., Ltd.) were housed according to the guidelines established by the Institutional Animal Care and Use Committee of Jilin University. All the *in vivo* assays were approved by this committee. A mouse pneumonia model of A. baumannii was established according to methods in a previous report (43). Cultures of the WT, Δ*ppk1*::Apr, and Δ*ppk1*::Apr/PJL02-*ppk1* strains of A. baumannii ATCC 17978 grown overnight were added to fresh LB broth at a ratio of 1:100 and cultured with shaking (180 rpm at 37°C). Logarithmic-growth-phase A. baumannii cells were collected at 6,000 rpm at 4°C for 8 min and washed with PBS twice. Next, 50-μL bacterial suspensions (1 × 10^9^ CFU/mouse) were inoculated intranasally to establish a mouse pneumonia model.

After infection for 48 h, lung bacterial loading and H&E experiments were conducted. The left lungs were excised for histopathological observation, weighed, homogenized, and centrifuged, and the supernatant was collected (10,000 rpm at 4°C for 10 min) to perform bacterial load counts. In addition, the levels of cytokines (IL-1β, IL-6, and TNF-α) in the lungs were determined by an ELISA (BioLegend, San Diego, CA, USA) according to the manufacturer’s instructions.

### Statistical analysis.

All assays were performed in triplicate. Data were analyzed by GraphPad Prism 5.0 using Student’s *t* tests. “**” (*P* < 0.01) indicates a highly significant difference, “*” (*P* < 0.05) indicates a significant difference, and ns indicates no significant differences. The data are presented as the means ± standard deviations (SD).

### Data availability.

The data that support the findings of this study are available from the corresponding author upon reasonable request.
