# Supplementary material for: Polyphosphate Kinase Is Required for the Processes of Virulence and Persistence in Acinetobacter baumannii
Source: Microbiol Spectr. 2022 Jul 5;10(4):e01230-22. doi: 10.1128/spectrum.01230-22 (PMC9430702; doi:10.1128/spectrum.01230-22)
Supplement: Supplemental file 1 — Supplemental material. Download spectrum.01230-22-s0001.pdf, PDF file, 0.3 MB [file spectrum.01230-22-s0001.pdf]

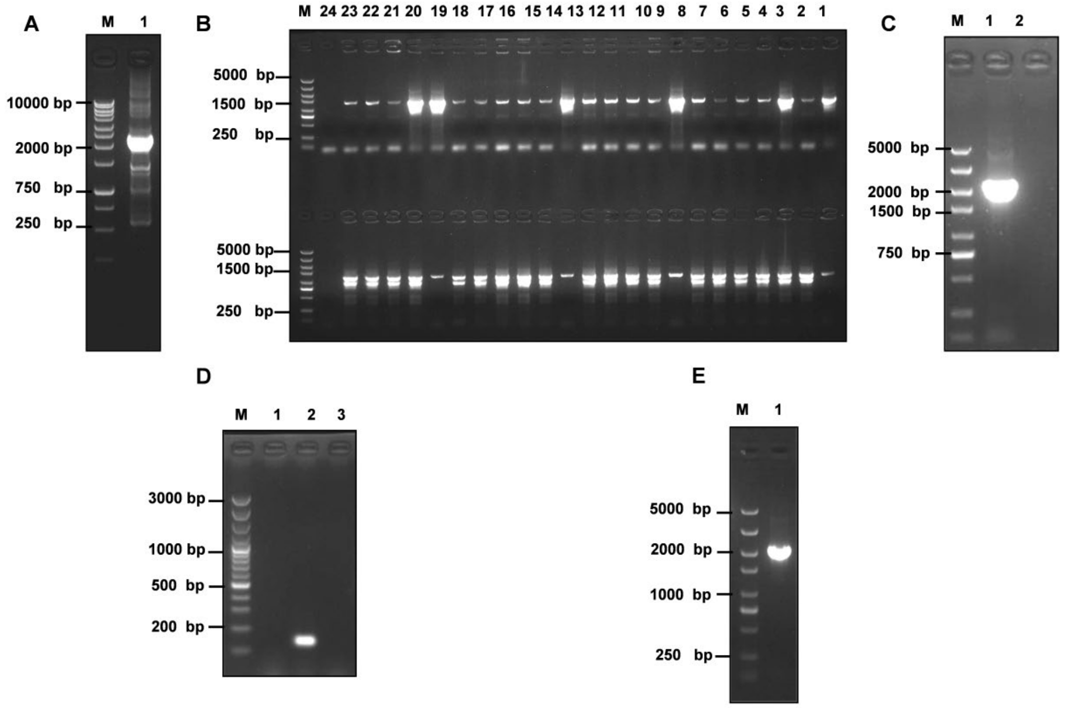

S1

Supplementary Figure 1 Generation of *ppk1*-knockout and complemented strains from *A. baumannii* ATCC 17978. (A) Nucleic acid electrophoresis map of marker (Line M) and apramycin resistance gene linked with the homologous recombination upstream and downstream DNA fragment of *ppk1* (Line 1). (B) The correct single exchanged Line 1, Line 3, Line 8, Line 13, Line 19, and Line 20 clone was screened by PCR using primers *ppk1*-out forward/reverse and *Apr*-seq forward/reverse and marker (Line M). (C) Nucleic acid electrophoresis maps of the apramycin resistance gene using primers *ppk1*-out F/*ppk1*-out R in Δ*ppk1*::Apr (Line 1), the negative control (Line 2), and the marker (Line M). (D) Nucleic acid electrophoresis maps of *ppk1* in Δ*ppk1*::Apr (Line 1) using primers *ppk1*-in F/*ppk1*-in R, the positive control (Line 2), and the negative control (Line 3). (E) Nucleic acid electrophoresis map of *ppk1* in *A. baumannii* ATCC 17978 using primers *ppk1*-BamHI-F/ *ppk1*-SalI-R (Line 1) and the marker (Line M).

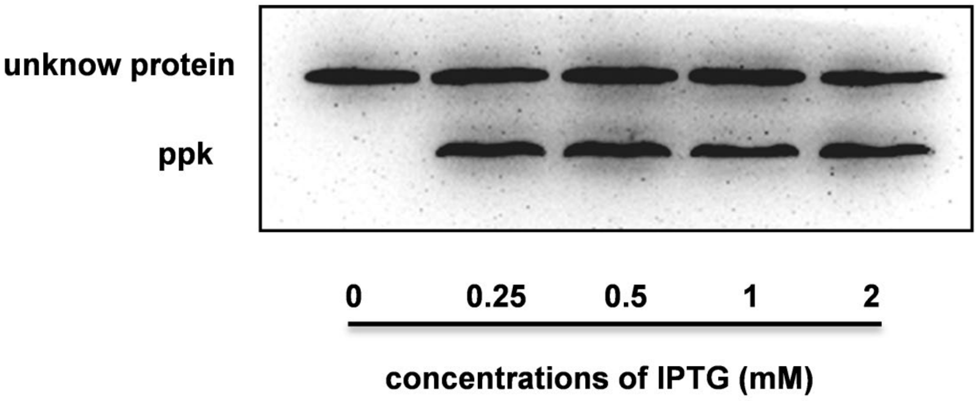

S2

Supplementary Figure 2 Western blot analysis of PPK1 in logarithmic phase Δ*ppk1*::Apr/PJL02-*ppk1* under induction with different concentrations of IPTG(0 mM, 0.25 mM, 0.5 mM, 1 mM and 2 mM).

Supplementary Table 1 Bacterial strains and plasmids used in this study.

Strains or plasmids	Relevant features	Source
A. baumannii ATCC 17978	Type strains	ATCC
Δ*ppk1*::Apr/PJL02-*ppk1*	Complementation strains of *ppk1*	This study
Δ*ppk1*::Apr	*ppk1* deficient strains	This study
pUC57-Apr	Source for *apr* gene	This study
pCVD442	A suicide plasmid for construction Δ*ppk1*::Apr	This study
E. coli β2155	*E. coli* strains for conjugation	This study
PJL02	Complementation plasmid of *ppk1*	(1)
E. coli WM3064	A donor strain for complementation of *ppk1*	This study

Supplementary Table 2 Primers used in this study

Primers name	Oligonucleotide (5'-3')
ppk1-up-F	CTCAAGTTTGGTTTGGTGGCGTAC
ppk1-up-R	GTCTAACCTAATCTCTTTCCAACC
ppk1-down-F	CAGGCGAAAAATTGCATAATGCTCAAAG
ppk1-down-R	GCTGGTAACAACTTTATAGCCTTTAGCC
ppk1-Apr-F	AGAGATTTTGGTTGGAAAGAGATTAGGTTAGAC
ppk1-Apr-R	CTTTGAGCATTATGCAATTTTTCGCCTGGGAATA
ppk1-out-F	GAAAGCCTTTATTGTTCGCG
ppk1-out-R	TGTGCAATTGCATAGTCATTGGC
ppk1-in-F	GTAAAGAACATCATGTCATGTTGTCTGC
ppk1-in-R	CCAAATCTTCAACGTCTTCGTTAAGTG
Apr-seq-F	CAGAGCAGATCATCTCTGATCCATTG
Apr-seq-R	CAATGGATCAGAGATGATCTGCTCTG
PJL02-out-F2	GGAATTGTGAGCGGATAACAATTTCAC
PJL 02-out-R2	GTGCTGCAAGGCGATTAAGTTG
ppk1-BamHI-F	GACGACAAGGGATCCATGAATACAGCGATTAC
ppk1-SalI-R	CTCGGTACCGTCGACTTATTTAAAAGTTTCTAATAAT

Reference

1. Jie J, Chu X, Li D, Luo ZA-O. 2021. A set of shuttle plasmids for gene expression in *Acinetobacter baumannii*. PLoS One 16:e0246918.
